# Identification of Microbial Strains via 2D Cross-Correlation of LC-MS Data

**DOI:** 10.1021/jasms.4c00101

**Published:** 2024-05-14

**Authors:** Tucker James Collins, Cathy Muste, Kevin G. Owens

**Affiliations:** Department of Chemistry, Drexel University, Philadelphia, Pennsylvania 19104, United States

**Keywords:** correlation analysis, micro-organism identification, LC-MS, proteomics, Orbitrap, strain identification

## Abstract

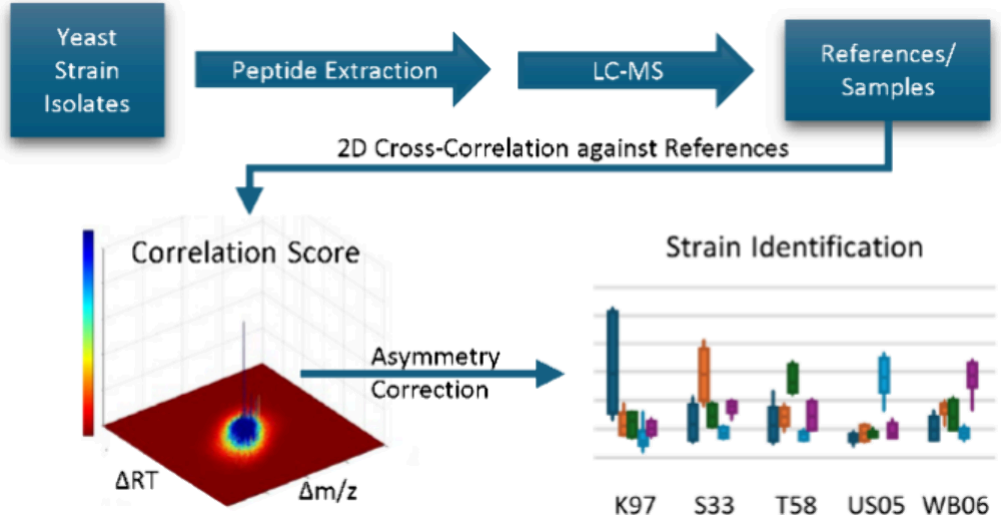

Mass spectrometry is commonly used in the identification of species present in microbial samples, but the high similarity in the peptide composition between strains of a single species has made analysis at the subspecies level challenging. Prior research in this area has employed methods such as Principal Component Analysis (PCA), the k-Nearest Neighbors’ (kNN) algorithm, and Pearson correlation. Previously, 1D cross-correlation of mass spectra has been shown to be useful in the classification of small molecule compounds as well as in the identification of peptide sequences via the SEQUEST algorithm and its variants. While direct application of cross-correlation to mass spectral data has been shown to aid in the identification of many other types of compounds, this type of analysis has not been demonstrated in the literature for the purpose of LC-MS based identification of microbial strains. A method of identifying microbial strains is presented here that applies the principle of 2D cross-correlation to LC-MS data. For a set of *N* = 30 yeast isolate samples representing 5 yeast strains (K-97, S-33, T-58, US-05, WB-06), high-resolution LC-MS-Orbitrap data were collected. Reference spectra were then generated for each strain from the combined data of each sample of that strain. Sample strains were then predicted by computing the 2D cross-correlation of each sample against the reference spectra, followed by application of correction factors measuring the asymmetry of the 2D correlation functions.

## Introduction

Mass spectrometry (MS) is a versatile analytical tool which may be used for the identification of compounds and can even serve as a means of identifying and sequencing peptides or classifying biological samples.^1−4^ MS-based methods such as liquid chromatography-electrospray ionization (ESI) and matrix-assisted laser desorption ionization (MALDI) MS enable the identification of numerous biomolecules in much less time than PCR-based identification.^5,6^ The dominant MS-based methods of identifying microbes presently generate libraries of peptide mass fingerprints (PMF) from MALDI-TOFMS data, which can then be compared against new samples analyzed by the same methods. Notable platforms designed on this premise are the Bruker, Inc. (Billerica, MA) MALDI Biotyper and the bioMérieux SA (Marcy-l’Étoile, France) VITEK MS, but these methods are primarily useful in identifying samples at the genus and species level only.^6−11^

The speed of the MALDI-based Bruker Biotyper and VITEK MS has been a significant advancement in microbiology laboratories by facilitating workflow automation and higher throughout analyses, but these instruments are not without limitations. A 2016 study by the National China Hospital Invasive Fungal Surveillance Net found that the efficacy of fungal species identification for 2500 isolates (41 total species) using both VITEK MS and the Biotyper is highly dependent on the availability of matching yeast isolates in the respective libraries, but could otherwise be effective at identifying well-represented species.^10^ Similarly, a 2014 meta-analysis of 33 published articles covering nearly 10 000 isolates of clinically pathogenic fungal species found that MALDI-TOF MS methods could be effective for identification at the species level for pathogenic species.^9^ Furthermore, a large collaborative study between 10 European institutions analyzed more than 1500 medically relevant yeast isolates (collected from human subjects) in order to compare different sample preparation methods for MALDI-TOF for use with either the Bruker database or an alternative database; by lowering the criteria for identification, a majority of samples could be identified at the species-level, though some sample preparation methods were deemed insufficient for yeast identification.^11^ These studies demonstrate that the utility of existing tools such as the Bruker Biotyper is great, but that it is often limited to species with clinical significance. While these methods may be suitable for identification of samples at the genus- and species-level, they are not generally suitable for microbial subspecies level identification.

In a 2020 article, Lasch et al. proposed a protocol for an LC-MS-based fingerprinting procedure, and the associated software package MicrobeMS,^12^ that combines data published in peptide databases (e.g., UniProtKB, SwissProt) to construct an in silico library of microbial peptide fingerprints.^13^ MicrobeMS eliminates the retention time dimension from the test data during the preprocessing steps, creating instead a mass spectrum that is analogous to the results attained directly via MALDI-based procedures. MicrobeMS scores new samples against the library spectra by calculating the interspectral distance between the observed and expected peptide masses, along with the respective intensities, weighting factors and observation frequency.^13^ While this method provides a novel means of utilizing LC-MS data to identify microbes, it is likewise sufficient only for genus and species level identification where a substantial amount of data has been imported or collected for the library.^13^ Similarly, in a 2014 study, Kern et al. attempted to use a Pearson correlation-based analysis with MALDI-TOFMS data to identify bacterial strains involved in the beer spoilage process, determining that the methods necessary to distinguish between different species is distinct from the goals involved with subspecies differentiation.^14,15^ While algorithms for species identification are optimized for quantifying the similarity between spectra, an algorithm for subspecies identification must be capable of identifying features that differentiate strains from each other. These goals are often in opposition since the higher intensity biomarkers are more likely to be species-specific biomarkers, while peaks that could differentiate between subspecies are often lower in intensity.^15,16^ While MicrobeMS, VITEK MS, and the Bruker Biotyper are all capable of genus and species level microbial identification, these algorithms are not designed for subspecies identification because of the limitations resulting from favoring peaks of greater relative intensity in sample identification.

Both Lasch & Kern make use of the Pearson’s product momentum correlation to quantify similarity between reference and test spectra, but this may not be the most optimal method of quantitative analysis for this data. The Pearson correlation coefficient quantifies the linear relationship between two data sets, whereas cross-correlation analysis measures the degree of similarity between two sets of data, making it potentially more appropriate for classification tasks of mass spectral data. Cross-correlation analysis has successfully been applied to identifying small-molecules from mass spectra when combined with a secondary correction factor that quantifies asymmetry in the correlation function,^3,4,17^ and has also had a prominent role in the field of proteomics as the basis of the scoring for SEQUEST and other related peptide-identification algorithms.^2,18,19^ In a 2021 report by You et al., a cross-correlation based algorithm was developed to analyze LC-MS chromatograms (using the Fourier transform in the retention time-domain) to identify small-molecule components of complex mixtures.^20^

In this initial report, an analytical approach for classification and identification of microbial subspecies analyzed by LC-MS against data for reference samples is proposed that scores samples via cross-correlation with a correction factor. Given the vast diversity among different strains of *S. cerevisiae*, it is a useful model organism in the development of a method of identifying microbial strains. The 12Mb genome of the S. *cerevisiae* species is well characterized in genome databases, with genomic data in the public domain covering more than 2000 distinct strains.^21^ Therefore, samples of five commercial strains of Brewer’s yeast have been cultured and analyzed by LC-MS to generate the microbial subspecies data for this report. Data were used to generate sets of reference and test spectra, which were subsequently compared by cross-correlation analysis to identify the strain of each yeast isolate sample against the reference samples. Results are presented for intensity normalized and non-normalized data, and several different correction factors are compared per the 1989 paper by Owens on cross-correlation of mass spectral data.^3^ The previously published work classifies compounds using the 1D correlation functions of 1D MS spectra while this study novelly computes the 2D cross-correlation functions for LC-MS data. The prior work by Owens also explores the use of correction factors that quantify asymmetry of the 1D correlation functions to improve sample classification. Several methods are demonstrated here where the 2D correlation functions are flattened with respect to the retention time domain and to the *m*/*z* domain, followed by computation of the 1D correction factors. Additionally, a 2D correction factor is computed for the 2D correlation function, and 1D correlations for mass spectra and chromatograms are analyzed separately.

## Computation and Properties of Correlation Functions

Correlation analysis may be computed in several different ways, depending on whether the input data are continuous or discrete, and the number of dimensions available. The universal equation for correlation analysis is given by eq 1, where the star operator indicates correlation, and the “*t*” subscripts indicate that the data being correlated are in the time-domain.^3,22^
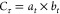
1

For continuous signals, the correlation function may be computed using eq 2 by integrating the product of two signals, *a*(t) and *b*(t), as a function of the displacement τ between them. In general, two signals are completely aligned in the correlation function where τ = 0.
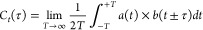
2

For discrete signals, this operation is equivalent to the calculation of the dot product of two signals as a function of the displacement τ between them as given in eq 3; the displacement τ is represented instead as *n*Δ*t*, where *n* is an integer multiple of a constant step-size.
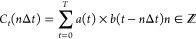
3

In essence, one signal is translated across the other, and the products of all overlapping elements of the matrices are summed together. The correlation function will therefore have a higher value when the signals have a substantial degree of overlap. While eqs 2 and 3 are given for a 1D correlation function, the correlation function of 2D signals may be computed by extending the operations above to include displacement with respect to an additional dimension.

Alternatively, a correlation function may be computed by multiplying the signals in the frequency domain. The time-domain signals (*a*_*t*_, *b*_*t*_) are converted to frequency-domain (*A*_*v*_, *B*_*v*_) by applying the Fourier transform  (in eqs 4 and 5).
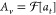
4
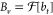
5

Multiplying the frequency-domain of one signal by the complex conjugate of the other, where *B̅*_*v*_ is the complex conjugate of *B*_ν_, gives the correlation function in the frequency domain (eq 6). The correlation function *C*_*t*_ is then found by taking the inverse Fourier transform  of the frequency-domain correlation function *C*_*v*_ (eq 7).^22^
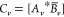
6
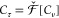
7

The Fourier transform-based calculation (eqs 4–7) may be applied to both continuous and discrete data and may be used to produce the *n*-dimensional correlation function for data with *n*-dimensions. Both the shift and the FT process generate the same correlation function when applied to discrete data sets, though the latter method is computationally much faster. Programs such as MATLAB often employ the fast Fourier transform (FFT) instead—this method capitalizes on the symmetry of the *n*-dimensional discrete Fourier transform matrices to improve computational efficiency.

Correlation analysis of mass spectral data has been demonstrated to be useful in classifying and identifying biomolecules, and here it is novelly applied to mass spectral chromatograms collected via LC-MS. Input data contain peak intensities as a function of retention time and mass-to-charge ratio. The output of the Fourier transform is then the inverse of each (RT^–1^ and *m*/*z*^–1^ in the Fourier-domain), such that the correlation function produced by eq 7 is returned to being a function of displacement τ with respect to RT and *m*/*z*.

Computation of the 2D correlation function of a sample set of data compared to a reference data set or other sample allows for a measure of similarity to be found to assess each sample. Software such as MATLAB, Maple and R all have built-in methods of applying cross-correlation and the Fourier transform; the native MATLAB functions **xcorr** and **xcorr2** compute the cross-correlation functions by the dot-product method (eq 3), where the inputs are vectors and matrices, respectively, for a 1D and 2D correlation.

In contrast to the previously explored methods, correlation analysis can be carried out in multiple dimensions through existing software such as MATLAB, opening the door to a new type of mass spectral fingerprint matching that utilizes both the peptide masses and retention time as features for classification. The application of a 2D cross-correlation based approach to LC-MS data to identify microbial strains is so far unexplored in the literature.

## Data Pre-Processing

The LC-MS peak lists must be fit into uniformly spaced arrays before computing the 2D cross-correlation function. This puts high resolution data in competition with the overall computing power required to calculate the correlation analysis function. At the cost of lowered resolution, it is practical to bin mass spectral peaks when constructing the uniform arrays.

Another common preprocessing step is to normalize the intensities of the mass spectral peaks—while mass spectral peaks are often normalized to the base peak, algorithms like SEQUEST normalize peaks in a somewhat more nuanced manner.^2,17,19,23^ Base peak normalization is explored in this report in comparison to results for non-normalized samples.

Correlation analysis is sensitive to several factors that need to be accounted for in the data preprocessing steps. Reference spectra containing a disproportionate number of peaks relative to other reference spectra will tend to have a greater correlation maximum, so preprocessing steps may need to reduce the total variability in the numbers of peaks contained in each reference spectrum. Two potential methods of doing this are to **sum** all values or to take the **max** value across a dimension; for a mass spectral chromatogram (peak intensities as a function of *m*/*z* and RT), applying the **sum** function with respect to *m*/*z* would generate the total ion chromatogram (intensity vs RT), while doing so with respect to *m*/*z* would instead generate a mass spectrum (intensity vs *m*/*z*). Applying the **max** function instead produces a base peak chromatogram when applied across the *m*/*z* dimension, where only the largest intensity peaks are retained with respect to the dimension being flattened, effectively reducing the number of peaks being accounted for to the size of the vector.

## Refining Correlation Analysis Results to Improve Classification

**Figure 1:**
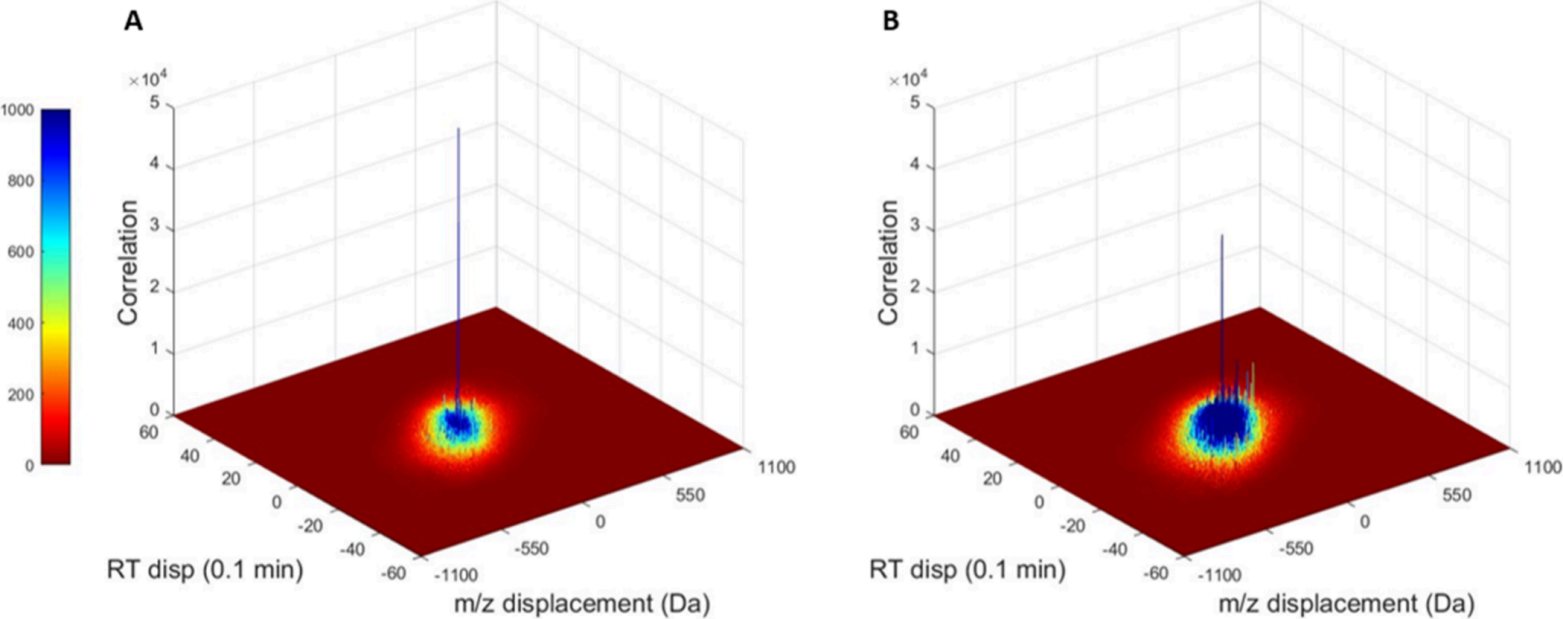
Examples of 2D Autocorrelation and Cross-Correlation functions. An autocorrelation is shown for S-33 yeast isolate at an incubation of 7-days (A); cross-correlation has been computed for yeast isolate samples of S-33 and T-58, each incubated for 7 days (B). The uncorrected correlation maxima for these comparisons are 4.94e+4 and 3.21e+4, respectively. Autocorrelations nearly always have correlation scores significantly greater than cross-correlations.

When both signals are identical, the generated “autocorrelation function” is totally symmetric with a maximum value at the locus corresponding to total signal alignment (τ = 0) (Figure 1). Otherwise, cross-correlation of nonidentical signals generate asymmetric correlation functions, and the maximum may drift from τ = 0 if the signals have greater alignment when offset from each other;^3^ this may occur if, for instance, there is an offset with respect to one or more dimensions (differences in retention time, *m*/*z* range, etc.). The raw score for sample identification is given by the correlation value at τ = 0.

Since the similarity of two input signals makes the correlation function more symmetric, quantifying the degree of asymmetry in the correlation function can also aid in differentiating samples. As demonstrated previously by Owens, several correction factors are presented here, which may be applied as a divisor to the raw similarity score to facilitate sample classification.^17^

Equations 8–10 provide methods of computing the asymmetry in a 1D correlation function of mass spectral data. The total sum correction is the mean absolute difference in the sums of correlation values on either side of τ = 0 (eq 8). By comparison, the sum difference 1 and 2 correction factors compare values in the correlation function with equal displacement from τ = 0; the former computes the mean of the differences between corresponding peaks (eq 9), while the latter compares the relative difference between corresponding peaks by dividing by the average of those values (eq 10). *C*_*t*,mid_ refers to the correlation value at τ = 0, while *C*_*t*,mid*±i*_ indicates the value of the correlation function at a corresponding distance *i* away from τ = 0.
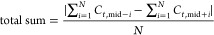
8
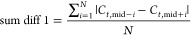
9
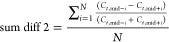
10

By contrast to the above, the imaginary correction is computed by summation of the imaginary components of the Fourier-domain of the correlation function *C*_*v*_, rather than from the time-domain of the correlation function (eq 11).
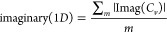
11

While the previously described correction factors have been applied to 1D cross-correlations of mass spectra, it is pertinent to adapt these other correction factors for a 2D correlation function of LC-MS data; eq 12 presents the equation for the 2D imaginary correction factor. 2D variants of eqs 8–10 would need to account for displacement with respect to both *i* and *j*.
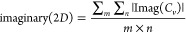
12

## Experimental Section

### Yeast Strains

Five commercial yeast strains (K-97, S-33, T-58, US-05, WB-06) were selected for the initial phase of this experiment, all purchased from Fermentis. The strains are Safale K-97 (Lot 201000250623), Safbrew S-33 (Lot 248071931802), Safbrew T-58 (Lot 102043500729), Safale US-05 Lot 296610461340), and Safbrew WB-06 (Lot 1863131752).

### Yeast Sample Preparation

For each yeast strain, 150 mg yeast was rehydrated (*n* = 3 preps each for 4 day incubation, *n* = 1 prep for 1-, 2-, and 7-day incubation times; 30 samples total) in 20 mL of 25 mg/mL glucose in tap water, then incubated in a sealed sterile scintillation vial with a sterile filter vent needle at room temperature in the dark. After incubation, samples were centrifuged for 5 min at 5000 rpm.

### Solid Phase Extraction

Solid phase extraction of the samples was conducted using a Waters C18 Sep-Pak Light (PN WAT023501, 130 mg sorbent) SPE cartridge with 0.1% formic acid in water as the weak eluent, and (60:40) acetonitrile: 0.1% formic acid in water as the strong eluent. The SPE cartridge was first conditioned with 2 mL strong eluent, followed by 2 mL weak eluent. The cartridge was then loaded with 10 mL yeast supernatant, washed with 2 mL weak eluent, and eluted with 1.0 mL strong eluent into an Eppendorf tube. Eluates were speed vacuumed to near-dryness and reconstituted with 200 μL 0.1% formic acid. Glucose control samples were also prepared using SPE (no yeast).

### Liquid Chromatography

LC/MS was conducted using a Thermo Vanquish UHPLC with a Waters Acquity CSH C18 column (150 × 2.1 mm, 1.7 μm). Five μL of SPE-extracted sample was injected onto the column (30 °C column temp; 0.2 mL/min flow rate), and separated by gradient elution (mobile phase A: 0.1% formic acid in water, mobile phase B: 0.1% formic acid in acetonitrile), solvent strength increased from 0–40% B from 0–60 min, followed by 12 min column wash at 95% mobile phase B (0.25 mL/min), and 18 min re-equilibration.

### Mass Spectrometry

Mass analysis was performed using a Thermo Q/Exactive Plus (Q/Orbitrap) in electrospray ionization positive mode; the first 3 min of eluate from the LC column was diverted to waste. Full MS was carried out from 400–2000 *m*/*z* at a resolution of 70 000.

### Initial MS Processing

All peaks were extracted using Thermo BioPharma Finder, at the default assigned MS threshold. The following peaks were then removed from the peak list: nontarget charge states (unassigned, 1+), unassignable monoisotopic mass, XIC abundance rank <3000, XIC peak area <10 000.

**Figure 2:**
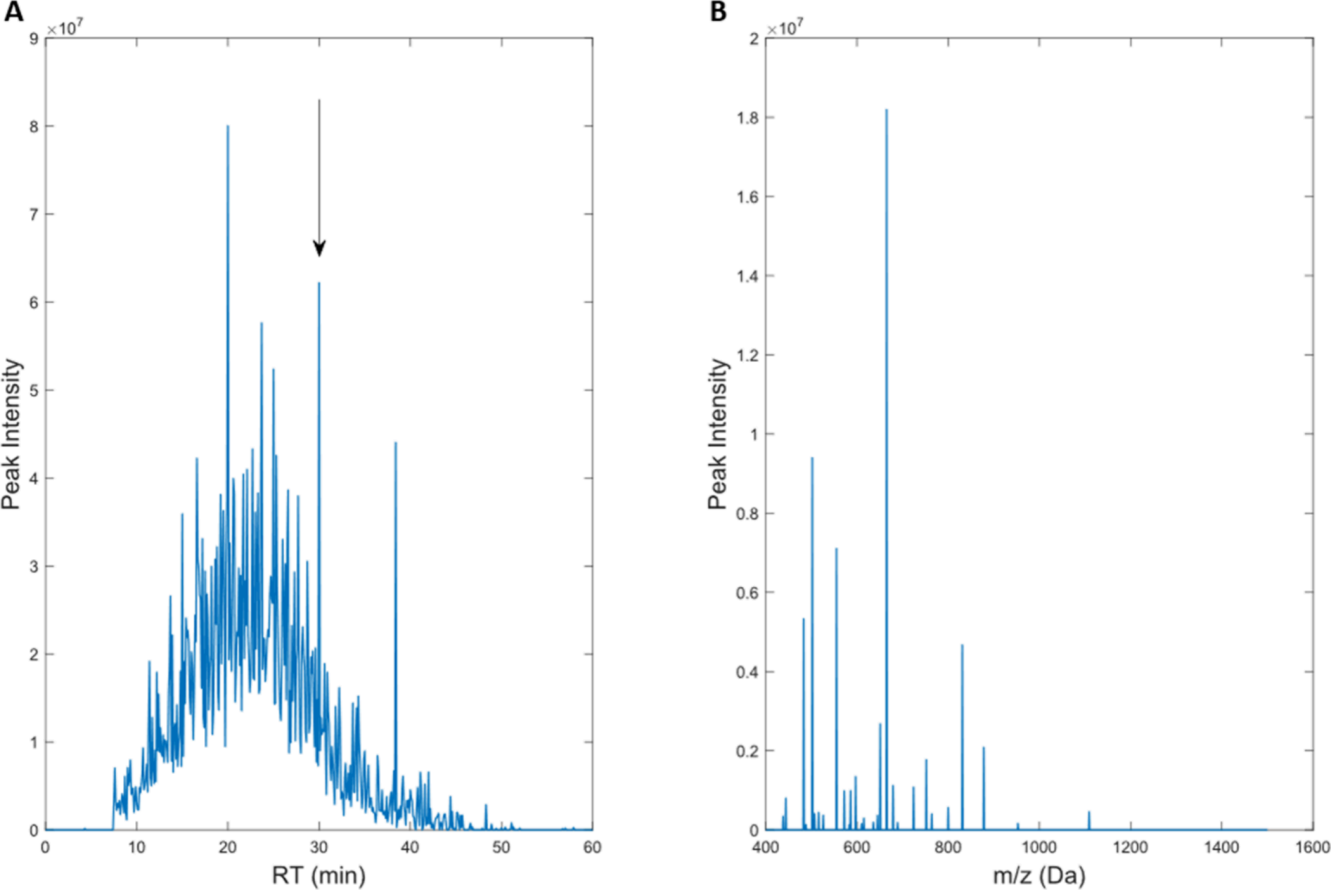
Total ion chromatogram (A) of peptides for a S-33 yeast sample rehydrated in a glucose solution and incubated for 7-days, and a mass spectrum (B) for the same the same sample at a retention time of 30.0 min.

After charge state deconvolution, peptide fragments were sequenced and identified from each mass spectrum; there are 30 peptide peak lists from the incubated yeast samples, and 6 additional mass spectra collected as controls.^24^ A typical example of the chromatogram and mass spectrum for a single sample are shown (Figure 2).

### Calculations

**Figure 3:**
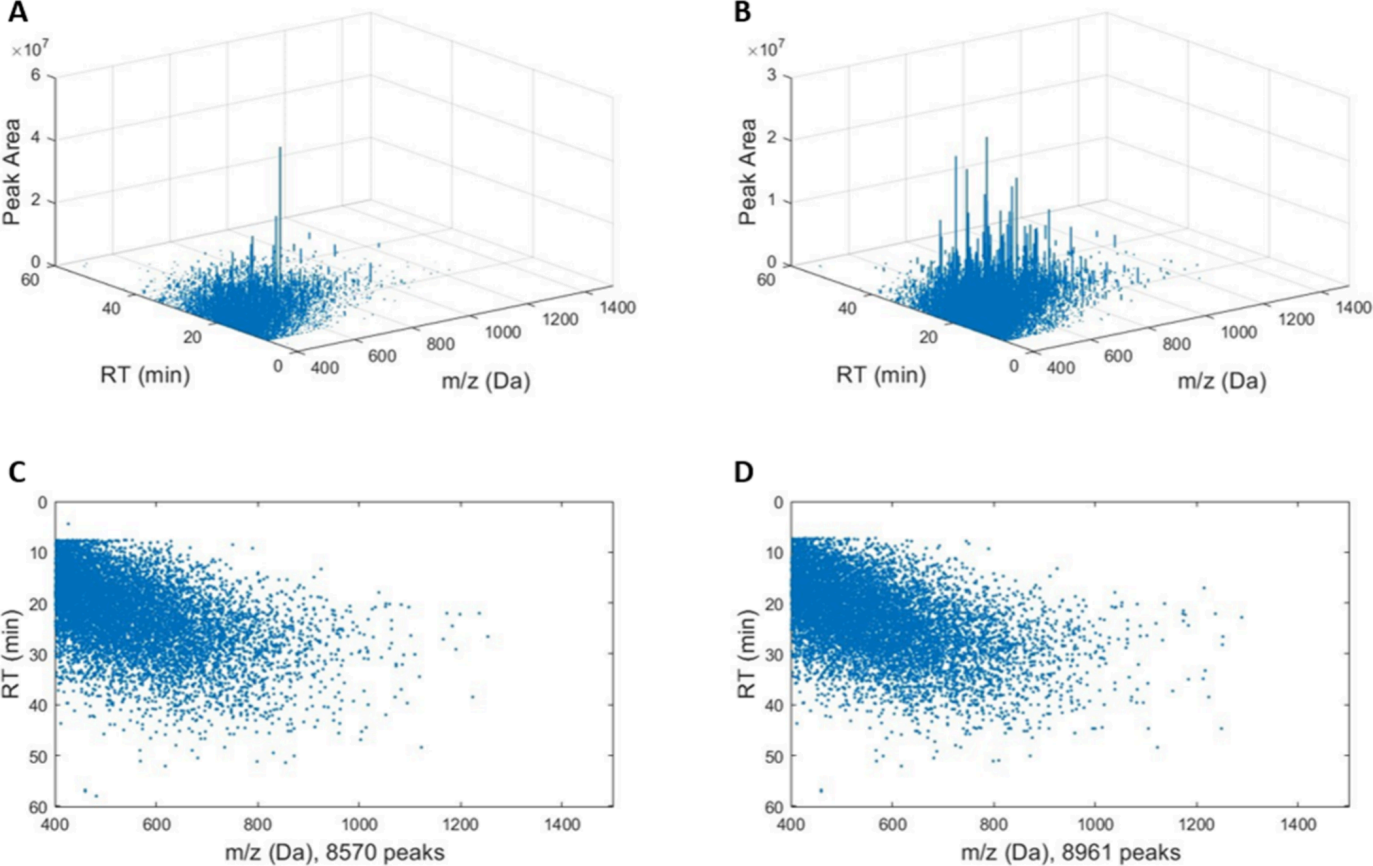
LC-MS data for yeast isolates of two yeast strains, S-33 (A,C) and T-58 (B,D) after 7-days incubation. Stem plots (A,B) show the peak data at its highest resolution for each sample. Sparsity plots of the same data (C,D) after binning are resolved to 0.1 min and 1 Da, demonstrating the locations of peaks with a nonzero intensity; the array value is the peak intensity. A reduction in the total number of peaks is observed due to the binning procedure; for S-33, nonzero peaks are reduced from 8981 → 8570 (95.4%); for T-58, nonzero peaks are reduced from 9406 → 8961 (95.3%).

All correlation computations have been carried out in MATLAB R2023a by MathWorks (Natick, MA, U.S.A.).^25^ To begin analyzing the data in MATLAB, the data from the peak lists were arranged into uniformly spaced arrays; this required bins to be defined with set dimensions. Retention times generally span from 4.00–54.00 min, while *m*/*z* values range from 400–1450 Da. Bins have been defined at present to have increments of 0.1 min and 1 Da, such that peak array Y has dimensions of 601 × 1101, corresponding to 0.0–60.0 min by 400–1500 *m*/*z*, respectively. Each locus Y_ij_ has corresponding ranges for retention time and *m*/*z*, and the value at Y_ij_ is the sum of the intensities of all peaks in that range (Figure 3). Most peaks are still well-resolved when binned at this resolution—across 30 3-D chromatograms, the total number of peaks is observed to be reduced by an average of 4.3 ± 1.1% (median: 4.5%). The peak arrays for each sample are duplicated with the intensities of Y_ij_ normalized to a base peak of 100.

### Reference Sample Creation

References were generated for each yeast sample using both the intensity-normalized peak arrays and the non-normalized peak arrays (10 references total, 2 references per strain). The non-normalized reference is generated by averaging the intensities for each of the 6 non-normalized peak arrays elementwise. Likewise, normalized references were generated by averaging the normalized peak arrays elementwise.

### Cross-Correlation of Yeast Data

Initial analyses were carried out without attempting to correct spectra due to retention time drift. Additionally, no peak selection or weighting has been introduced so that an assessment could be made of the correlation analysis method on its own.

While the **xcorr** and **xcorr2** functions allow for computation of the 2D correlation function, custom functions **FTcorr1** and **FTcorr** were generated to compute the correlation function using the fast Fourier transform instead (eqs 4–7), facilitating computation of the imaginary correction factors, (eqs 11, 12).

Sample spectra are compared against the reference spectra by computing the 2D correlation function via the custom **FTcorr** function in MATLAB. The function creates a correlation array ***A*** with dimensions (*m*_1_ + *m*_2_ – 1) × (*n*_1_ + *n*_2_ – 1), where each locus gives the correlation value with respect to displacement in retention time and *m*/*z*.

To assess the initial capacity for this method to identify strains using correlation analysis, and with these correction factors, a set of reference samples were created by averaging 6 spectra from each strain together, to create a small library of 5 reference spectra (one for each strain studied). Cross-correlation functions and correction factors were computed between each of the reference spectra with each of the 1, 2, 4, and 7-day yeast samples (30 spectra total).

### Strain Identification

Three analytical workflows are assessed here, with consideration of how to apply 1D correction factors to LC-MS data. All methods were applied to the non-normalized and intensity-normalized samples. Reference spectra were treated the same as the test samples for each method. Correction factors are applied as scalar divisors of the correlation value at τ = 0 to generate the final scores for each sample; the predicted strain for each sample is the reference strain with the highest score for the given method. *Methods 1* & *2* involve the 2D cross-correlation, while *Method 3* uses the 1D cross-correlation after first reducing the LC-MS data to either mass spectral or chromatographic data.

#### Method 1

The 2D cross-correlation function is computed by **FTcorr** between each LC-MS sample data set and each of the 5 reference spectra. The correlation function is then flattened with respect to either RT or *m*/*z* to generate a *m*/*z*-domain or RT-domain correlation function, respectively. Correction factors are subsequently computed using eqs 8–10. See Tables S1–S16 in the Supporting Information (SI).

#### Method 2

The 2D cross-correlation function is computed by **FTcorr** between each LC-MS sample data set and each of the 5 reference spectra. The raw score is the τ = 0 value, and the 2D imaginary correction (eq 12) is computed from the Fourier domain of the 2D cross-correlation function. See Tables S17–S20 in the SI.

#### Method 3

First, the LC-MS data are reduced to either mass spectra or chromatogram only data using the **max** or **sum functions**. Mass spectra were generated by taking the maximum peak intensity for each *m*/*z* across all RTs (MS, max) or by summation of all peak intensities for each *m*/*z* across all RTs (MS, sum). Base peak chromatograms (BPC) and total ion chromatograms (TIC) were generated by taking the maximum peak intensity for each RT across all *m*/*z* values (BPC, max) or by summation of all peak intensities for each RT across all *m*/*z* values (TIC, sum). The MS, BPCs, and TICs were then analyzed by 1D cross-correlation (**FTcorr1**), followed by calculation and application of correction factors using eqs 8–11. See Tables S21–S44 in the SI.

## Results and Discussion

**Figure 4:**
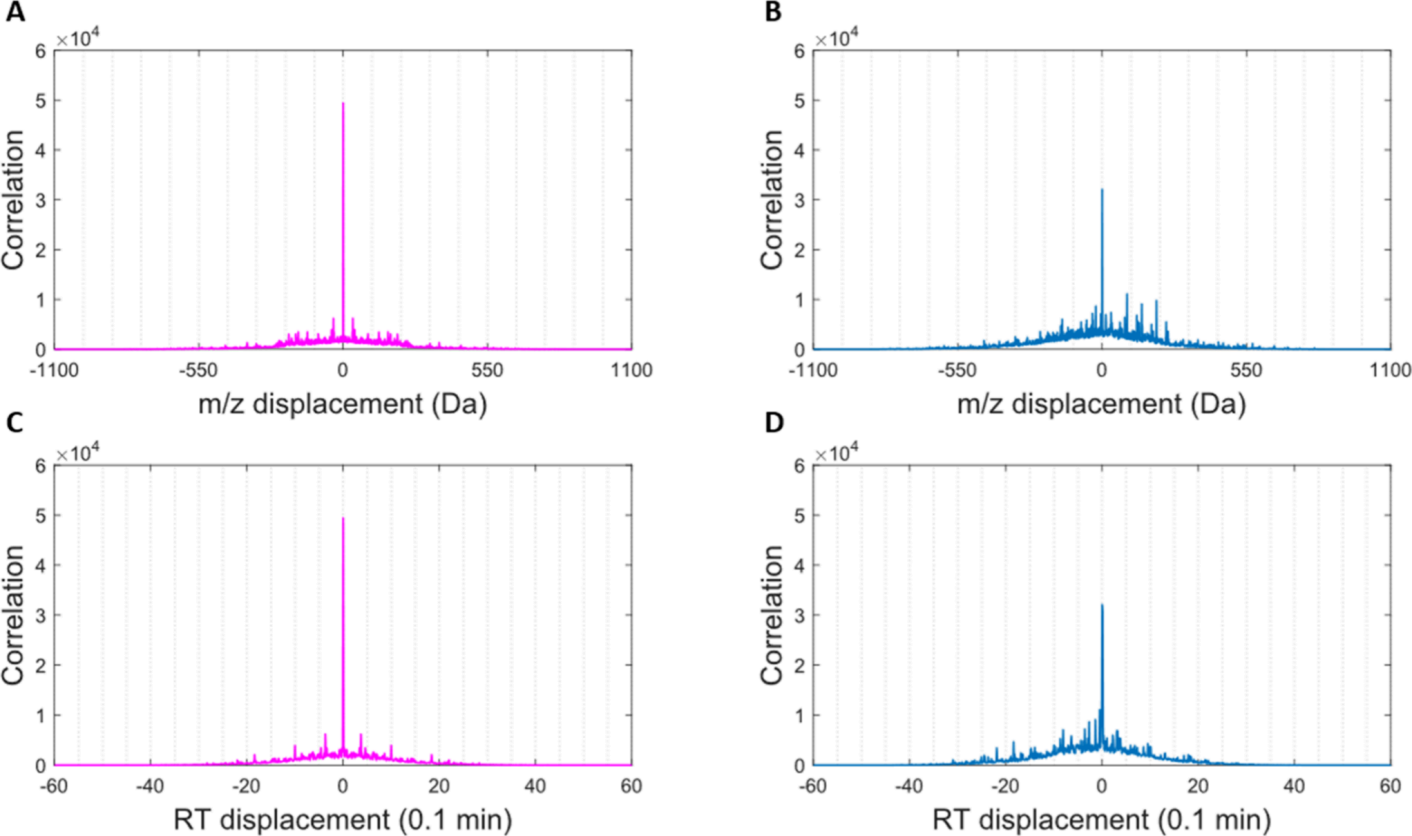
2D correlation functions flattened with respect to the *m***/***z*- and RT-domains for the autocorrelation of 7-day S-33 yeast isolate, and the cross-correlation of 7-day yeast isolates of S-33& T-58. Autocorrelation functions are symmetric across τ = 0 with respect to *m*/*z* (A) and RT (C), while cross-correlation functions (B, D) have asymmetry when displaced positively or negatively from τ = 0.

While Figure 1 shows examples of 2D auto- and cross-correlations, Figure 4 shows the same 2D correlations flattened with respect to the *m*/*z*- and RT-domains to further demonstrate the asymmetry of cross correlations as compared to autocorrelation functions. The correlation maximum is typically observed in the range of ±4 channels of *C*_*t*,mid_ (τ = 0) in the RT-domain for both non-normalized and normalized samples; when compared against the reference of the matching strain, 26 and 27 of the correlation maxima (of *N* = 30) were located at *C*_*t*,mid_ for non-normalized and normalized samples, respectively, which is consistent with the effect of retention time drift. As expected, no drift is observed in the correlation maximum with respect to displacement in *m*/*z* due to the high precision in the Orbitrap data. Furthermore, in agreement with prior research on the application of correlation analysis for mass spectral-based identifications, autocorrelations are observed to be symmetric along both domains (Figure 4A,C), while cross-correlations are asymmetric with respect to both the *m*/*z*- and RT-domains of the 2D cross-correlation (Figure 4B,D).

Table 1 presents the uncorrected 2D cross-correlation scores (τ = 0) for each of the intensity normalized sample data sets correlated with the five reference strain data sets (also intensity normalized) calculated using *Method 2*. The largest value in each row (bolded) predicts the identity of the sample. Comparing labels in the leftmost to rightmost column, it can be clearly seen that 27 of the 30 samples are identified to the strain level correctly.

**Table 1:** Scores for the 2D Cross-Correlation of Intensity-Normalized LC-MS Spectra with No Correction Factor^a^

		reference strain					
strain	inc. (day)	K97	S33	T58	US05	WB06	prediction
K97	1	**37647**	11280	9734	3408	13032	K97
K97	2	**29026**	10468	18825	12307	16383	K97
K97	4	**89183**	21839	30402	13624	24574	K97
K97	4	**79178**	31047	33243	19528	29889	K97
K97	4	**90123**	23892	32248	16003	27184	K97
K97	7	**44858**	13509	16442	41808	16134	K97
S33	1	7113	16843	11141	8997	**18036**	WB06
S33	2	11300	**26907**	16224	17150	26251	S33
S33	4	26476	**31829**	20006	10834	22506	S33
S33	4	**31584**	27775	23013	12355	25841	K97
S33	4	27187	**31330**	22788	15686	23730	S33
S33	7	8375	**14882**	11038	13510	14260	S33
T58	1	7800	12505	**26510**	9340	13564	T58
T58	2	11363	13278	**29649**	15080	13729	T58
T58	4	41346	20595	**41682**	12224	29304	T58
T58	4	31733	23794	**39896**	16533	30606	T58
T58	4	28937	20890	**44677**	16898	32370	T58
T58	7	19714	13149	**42793**	23420	18451	T58
US05	1	31076	18761	18965	**90350**	20494	US05
US05	2	25574	16954	21721	**93171**	18221	US05
US05	4	14960	13443	12949	**46359**	17913	US05
US05	4	14085	14886	16002	**43898**	21636	US05
US05	4	14821	8412	15122	**55816**	17340	US05
US05	7	6162	6075	8736	**23536**	8954	US05
WB06	1	11914	21007	14180	11078	**38670**	WB06
WB06	2	15413	27477	18721	16268	**48708**	WB06
WB06	4	33996	24252	32921	16661	**50393**	WB06
WB06	4	30363	24582	33316	21893	**44807**	WB06
WB06	4	24404	20016	24933	18783	**43925**	WB06
WB06	7	11107	13290	13954	**19875**	25184	US05

a The maximum score for each sample is bolded. Inc. = Incubation duration.

Table 2 shows the same results after applying the 2D imaginary correction (eq 12) to the data shown in Table 1. Again, the largest value in each row is bolded, and is used to predict the identity of the strain in the sample. In this case all 30 of the data sets are predicted correctly.

**Table 2:** Scores for the 2D Cross-Correlation of Intensity-Normalized LC-MS Spectra after Applying the 2D Imaginary Correction^a^

		reference strain					
strain	inc. (day)	K97	S33	T58	US05	WB06	prediction
K97	1	**1.0637**	0.4470	0.3294	0.0863	0.4100	K97
K97	2	**0.6650**	0.3922	0.5596	0.2810	0.4693	K97
K97	4	**2.6290**	0.6461	0.7382	0.2539	0.5454	K97
K97	4	**1.8813**	0.9440	0.7991	0.3502	0.6835	K97
K97	4	**2.5445**	0.7076	0.7879	0.2950	0.6030	K97
K97	7	**0.8082**	0.3811	0.3735	0.7966	0.3510	K97
S33	1	0.2577	**1.0347**	0.5278	0.3417	0.8333	S33
S33	2	0.3178	**1.3652**	0.6022	0.5185	0.9671	S33
S33	4	0.8737	**2.0560**	0.8308	0.3274	0.8861	S33
S33	4	1.0644	**1.5701**	0.9644	0.3847	1.0088	S33
S33	4	0.8523	**1.8615**	0.9094	0.4618	0.9134	S33
S33	7	0.3182	**0.8978**	0.5445	0.5485	0.6661	S33
T58	1	0.2337	0.6101	**1.1035**	0.2944	0.5049	T58
T58	2	0.3145	0.6001	**1.1523**	0.4417	0.4738	T58
T58	4	1.1627	0.8465	**1.6032**	0.3117	0.9317	T58
T58	4	0.7901	0.9400	**1.3998**	0.3942	0.9636	T58
T58	4	0.7060	0.8199	**1.6911**	0.4194	1.0283	T58
T58	7	0.4085	0.4459	**1.2394**	0.4848	0.4785	T58
US05	1	0.4553	0.4381	0.3587	**1.7170**	0.3698	US05
US05	2	0.3877	0.4116	0.4265	**1.8375**	0.3383	US05
US05	4	0.3863	0.5493	0.4331	**1.4267**	0.5746	US05
US05	4	0.3466	0.5872	0.5151	**1.2564**	0.6706	US05
US05	4	0.3129	0.2731	0.4119	**1.3898**	0.4495	US05
US05	7	0.2003	0.3064	0.3649	**0.8169**	0.3486	US05
WB06	1	0.3013	0.8984	0.4745	0.2946	**1.3718**	WB06
WB06	2	0.3286	0.9992	0.5272	0.3640	**1.4822**	WB06
WB06	4	0.8156	0.9151	1.0577	0.3866	**1.7033**	WB06
WB06	4	0.6722	0.8695	0.9872	0.4799	**1.3902**	WB06
WB06	4	0.6283	0.8233	0.8604	0.4893	**1.6312**	WB06
WB06	7	0.3022	0.5611	0.4856	0.5598	**0.8287**	WB06

a The maximum score for each sample is bolded. Inc. = Incubation duration.

Rather than showing all of the detailed cross-correlation results (which are available as Tables S1–S44 in the SI), Table 3 summarizes the number of successfully classified samples obtained using *Methods 1 & 2* on both intensity normalized and non-normalized data sets. Using *Method 1*, the Sum Diff. One and Sum Diff. Two correction factors each correctly classified 96.7% of the test samples (*N* = 30) when classifying samples using the *m*/*z*-domain of the correlation function, regardless of normalization. Using the uncorrected τ = 0 value of the 2D-correlation alone yielded 22 correctly for the non-normalized samples compared to 28 correct with normalized samples. Notably, the uncorrected scores for the 2D-correlation are consistent with the results from flattening the 2D-correlation to the RT-domain. The correlation maximum of some samples is displaced from τ = 0 in the RT-domain, resulting in the τ = 0 value in the *m*/*z* domain being eclipsed by the correlation maximum, thereby increasing the uncorrected score in the *m*/*z*-domain. By contrast, the 2D Imaginary correction computed directly from the Fourier domain of the 2D-correlation function (*Method 2*) correctly identified 28 samples with non-normalized intensities, and all 30 samples with normalized intensities (and as shown in Table 2). Overall, normalizing the peak intensities prior to correlation analysis typically improved the number of samples correctly identified. Additionally, samples are more often correctly matched based on corrected scores derived from the *m*/*z*-domain versus the RT-domain, which is consistent with the observation of drift in the RT-domain.

**Table 3:** Accuracy of Sample Identifications from τ = 0 of the 2D Cross-Correlation of LC-MS Spectra Using Different Correction Factors

	non-normalized		normalized	
correction factor	*Method 1*: flattened 2D, *m*/*z*-domain			
none	20	66.7%	26	86.7%
total sum	22	73.3%	19	63.3%
sum difference 1	29	96.7%	29	96.7%
sum difference 2	29	96.7%	29	96.7%
correction factor	*Method 1*: flattened 2D, RT-domain			
none	22	73.3%	28	93.3%
total sum	14	46.7%	13	43.3%
sum difference 1	27	90.0%	28	93.3%
sum difference 2	25	83.3%	25	83.3%
correction factor	*Method 2*: 2D cross-correlation			
none, 2D	22	73.3%	28	93.3%
imaginary	28	93.3%	30	100.0%

Classification results for samples analyzed by cross-correlation of 1D Mass spectra, per *Method 3*, are presented in Table 4. Mass spectra (*N* = 30) were generated using the **max** or **sum** function prior to cross-correlation. Applying the imaginary correction to the 1D-correlation function correctly identified the strain of 29 non-normalized samples, and 29 normalized samples for mass spectra computed by **max**, but just 25 samples when the mass spectra were computed by **sum** for both non-normalized and normalized samples. Identifications using the imaginary correction factor tend to be more accurate than using the other correction factors. The **max** function typically resulted in a greater number of correctly identified strains compared to samples analyzed by **sum**; in contrast to the data shown in Table 3, normalizing peak intensities did not improve classification success using mass spectra.

**Table 4:** Accuracy of Sample Identifications from τ = 0 of the 1D Cross-Correlation of MS Spectra Using Different Correction Factors

	MS, max				MS, sum			
correction factor	non-normalized		normalized		non-normalized		normalized	
none	17	56.7%	17	56.7%	12	40.0%	12	40.0%
total sum	16	53.3%	16	53.3%	16	53.3%	16	53.3%
sum difference 1	24	80.0%	24	80.0%	16	53.3%	16	53.3%
sum difference 2	24	80.0%	24	80.0%	15	50.0%	15	50.0%
imaginary 1D	29	96.7%	29	96.7%	25	83.3%	25	83.3%

Classification results for samples analyzed by cross-correlation of 1D chromatograms (*N* = 30), per *Method 3*, are presented in Table 5. Base peak chromatograms (BPC) were computed from the LC-MS data by taking the maximum intensity peak for each RT (**max**), while the total ion chromatograms (TIC) are created by summing the intensities of all peaks at each RT (**sum**). Applying the imaginary correction to the 1D-correlation functions correctly identified the strain of 25 non-normalized samples and 25 normalized samples for BPC samples, but correctly identified only 16 TIC samples for both non-normalized and normalized samples; the best correction factors for TICs were the Sum Difference 1 & Sum Difference 2 at 17 correctly identified strains. Comparing the results presented in Tables 4 and 5, cross-correlation of chromatograms is the least reliable method of classification explored here, although this is likely due to the presence of yet uncorrected drift on the retention time axis.

**Table 5:** Accuracy of Sample Identifications from τ = 0 of the 1D Cross-Correlation of Base Peak (BPC) and Total Ion (TIC) Chromatograms Using Different Correction Factors

	BPC, max				TIC, sum			
correction factor	non-normalized		normalized		non-normalized		normalized	
none	20	66.7%	20	66.7%	12	40.0%	12	40.0%
total sum	15	50.0%	15	50.0%	16	53.3%	16	53.3%
sum difference 1	21	70.0%	21	70.0%	17	56.7%	17	56.7%
sum difference 2	18	60.0%	18	60.0%	17	56.7%	17	56.7%
imaginary 1D	25	83.3%	25	83.3%	16	53.3%	16	53.3%

## Conclusions

The results presented here demonstrate an analytical pathway for classification of microbial samples at the strain level is achievable through a 2D cross-correlation based approach to compare spectral similarities, followed by application of a correction factor to differentiate samples based on the asymmetry in the correlation function. Such classification has been carried out here without any biomarker selection process, which present a host of possibilities for ways in which spectral libraries may be refined to improve discrimination of distinct microbial species and strains.

Classification by any method that was based on the RT-domain of the correlation functions, or based on correlation of chromatograms, were largely inferior to methods focused on the *m*/*z* domain. This is hypothesized to be a result of uncorrected retention time drift between spectra, leading to greater variability in the RT-domain, and it is anticipated that correction of retention time drift in the future may make RT a more significant contributor to the overall classification success. In spite of this fault, the classification success (25, *N* = 30) for the 1D imaginary correction on the BPCs is nevertheless a testament to the overall benefits of the cross-correlation based classification algorithm.

Across 150 correlation functions between the 5 reference spectra and each of the 30 samples, deviation between the τ = 0 and correlation maxima were relatively rare when the strain was correct (10–13.3%, *N* = 30), while deviation was common when the strain did not match (58.3%, *N* = 120). This discrepancy suggests that the number of samples with deviations due to RT drift specifically may be relatively limited overall, while supporting the assertion that this method is beneficial for classification purposes. Nevertheless, correction of RT drift is expected to constitute a significant improvement for the method.

The accuracy of the analytical method here should also be noted as it has been carried out after significantly decreasing the resolution of data in the *m*/*z* domain from 0.0001 to 1 Da via binning with no apparent loss in classification ability. The potential to identify microbial strains from lower resolution mass spectral data would significantly enhance the existing methods of strain analysis, and make this a more universally available technique. Future experiments will compare sample classification using data collected from a higher resolution LC-MS-Orbitrap to a lower resolution LC-MS-QQQ instrument.

The proteins analyzed in this experiment were secreted proteins from yeast isolates, though many shotgun proteomics protocols employ tryptic digestion.^13,23,26,27^ Application of tryptic digestion to the sample preparation protocol would be a reasonable measure to explore in future work. The peptides observed in these samples were in the range of 400–1500 *m*/*z*, but this range could easily be adapted to the typical *m*/*z* range of peptides in tryptic digests.

Application of the 2D imaginary correction factor directly to the 2D correlation function was the most successful method used, correctly identifying the strains of all 30 samples. While the Sum Diff. One and Two correction factors worked well in *Method 1*, adapting all the other correction factors (eqs 8–10) to be used directly with the 2D correlation may also improve the results over applying them to flattened versions of the 2D correlation. Across all methods of analysis, the imaginary correction gave better results than the other correction factors; notably, it is also the only true 2D correction factor that was applied here. The results of applying the Sum Diff One and Two corrections also suggest that they may be reasonable alternatives to the imaginary correction once the retention time drift is addressed.

In future work, the method will be applied to a larger set of microbial strains, in addition to the further comparison of the use of different instruments for collecting the mass spectral data. Preprocessing methods will be further developed to account for retention time drift, and options for biomarker selection will be explored. Where the only true 2D correction factor applied here to the 2D correlation function was the imaginary correction factor, each of the other 1D correction factors will be adapted for 2D analysis, as well.

## Supporting Information Available

Tables of all uncorrected and corrected correlation results obtained using methods 1–3 (XLSX)
